# Communication and Community Involvement to Support Risk Governance

**DOI:** 10.3390/ijerph16224356

**Published:** 2019-11-08

**Authors:** Liliana Cori, Fabrizio Bianchi, Mario Sprovieri, Angela Cuttitta, Silvia Ruggieri, Anna Lisa Alessi, Girolama Biondo, Francesca Gorini

**Affiliations:** 1 Institute of Clinical Physiology, National Research Council, 56124 Pisa, Italy; fabrizio.bianchi@ifc.cnr.it (F.B.); fgorini@ifc.cnr.it (F.G.); 2 Institute for the Study of Anthropic Impacts and Sustainability in the Marine Environment, National Research Council, 00146 Rome, Italy; mario.sprovieri@cnr.it (M.S.); angela.cuttitta@ias.cnr.it (A.C.); annalisa.alessi@ias.cnr.it (A.L.A.); girolama.biondo@ias.cnr.it (G.B.); 3 Institute for Biomedical Research and Innovation, National Research Council, 90146 Palermo, Italy; silvia.ruggieri@irib.cnr.it

**Keywords:** environment and health, high risk areas, contaminated sites, risk governance, human biomonitoring, environmental monitoring, risk communication, training and education

## Abstract

In past years, communication activities have become increasingly important in the environment and health domain, considering the concurrent developments of social media and scientific citizenship that contributed changes in legislation and culture. Communication is particularly crucial where an environmental hazard is present, as in the case of high risk environmental and health risk areas. The project “International Center of Advanced Study in Environment, Ecosystem and Human Health” (CISAS), carried out by the Italian National Research Council, covers multiple research activities, from ecology to biology and medical sciences, from epidemiology to social sciences and communication. Three different studies based on human biomonitoring and a birth cohort study are currently in progress in the project locations, together with studies on the environmental fate of pollutants. A clear, accurate and respectful communication of study protocols and results represents a priority to produce comprehensible information available for policy makers, citizens, and stakeholders. This paper describes the multiple external and internal communication activities planned in the framework of the CISAS project as an example of promotion of knowledge in the society at large and improvement of risk management in the environmental health domain.

## 1. Introduction

The attention of the scientific community towards communication activities grew in recent years: the availability of research data and results is considered a key feature, managed through open access publications, to improve the dialogue with the group of peers; the publication of research protocols is a mandatory component of research, especially when issues of general interest are at stake. Finally, transparent results can be crucial to enhance the results significance, if they are shared with the stakeholders and transformed into policy recommendations [1,2].

Considering the concurrent developments of social media and scientific citizenship that contributed to changes in legislation and culture, the role of each stakeholder in the communication arena can be questioned, it is effectively questioned, and is barely predictable [3]. In particular, in the environment and health domain which is covered in the present article, citizens increasingly demand more and more information to contribute to their individual and collective protection and prevention. This may help policy makers in addressing citizen’s concerns and balance them together with the scientific evidence [4].

In the case of studies based on individual markers of exposure or early effect (human biomonitoring, HBM), specific ethical requirements regulate the protection of volunteers’ privacy; the dissemination and communication of results at different levels requires further attention [5]. Moreover, the 1998 Aarhus Convention on Access to Information, Public Participation in Decision-making and Access to Justice in Environmental Matters have been implemented in many government policies, thus guaranteeing the respect of a fundamental right [6].

Planning communication activities requires proper attention, based on knowledge of social and historical features of the communities involved. Communication is particularly important where a recognized environmental hazard is present, a public alarm is raised, a clear understanding of the risk is lacking and controversies arise among the stakeholders [7]. This happens in the three areas in Italy where systematic research activities regarding the cycle of environmental pollution and the pathways of contaminants in environment, food, human body are in progress in the framework of the “International Centre of Advanced Study in Environment, Ecosystem and Human Health” (CISAS) project of the National Research Council [8]. The CISAS project, in addition to research on environment, ecology and food chain, is carrying out three different epidemiological studies based on human biomonitoring (HBM) and a birth cohort survey in the contaminated sites of Augusta and Milazzo in Sicily and Crotone in Calabria, southern Italy. The communities involved have a total population of about 400,000 citizens, and because of serious environment and health issues, arouse attention at regional and national level. The number of adult donors involved in the three epidemiological studies is estimated at 1500, and the size of the birth cohort is 450 child–mother diads during the first year of recruitment [9].

The CISAS project embraces multidisciplinary activities, carried out by four Institutes of the National Research Council (NRC), in collaboration with scientific and regulatory bodies. The core CISAS group involves 153 researchers or technicians (84 from NRC institutes and 69 from other institutions). Their competencies include ecology, biology, medical sciences, epidemiology, social sciences, and communication. Among them, a group of experts was directly involved in communication activities, produced a communication plan and coordinated the activities illustrated in the present article. Careful planning was especially dedicated to project presentation and results communication, to identify receivers and suitable study instruments. The presentation of the information is crucial to facilitate a positive development of a multiplicity of different activities including interviews, collection of human specimens, involvement of schools and environmental monitoring. The process of elaboration and analysis of the results and their presentation is a specific research issue: for example, the sampling of water, sediments and fish in high-risk areas can disclose the existence of heavy contamination, requiring urgent interventions for public health protection. In the case of HBM the results should contribute to addressing reclamation activities providing a list of priority contaminants in the interested areas. A suitable communication strategy can contribute information useful for prevention and protection to the relevant decision makers. 

Long-term education activities constitute a strategic interest for the CISAS research group, to call citizen for a leading role in peer education and in influencing the community at large, and to support the growth of scientific citizenship. The interest in carrying out training and communication activities in schools is reinforced by high unemployment rates in the study areas and more generally in Southern Italy [10]; as a consequence, school teachers are key players in the educational component of the project.

The objective of this paper is to provide an overview of the communication issues involved in a multidisciplinary project dealing with environment and health in high risk areas, to inform the scientific community and the stakeholders about means and project activities that are in progress, first results and expected outcomes. Those activities are constitutive components of risk governance practices, as conceived by the International Risk Governance Council (IRGC) using an approach that integrates an analysis of the context, the specific risk characteristics and the multiplicity of stakeholders acting in the governance arena [11]. The European Commission provides a robust definition of governance in general, indicating its foundation principles, that are openness, participation, responsibility, efficacy, coherence [12]; risk governance is also examined in details by several EU documents, and stakeholder participation is required by EU in policy activation [13].

## 2. Materials and Methods

### 2.1. Communication Plan

In planning communication activities, the working group collected documents and available data together with information from exploratory fieldwork and meetings dedicated to share information with social actors and nongovernmental organizations (NGOs). Traditional and modern communication tools are available to perform different activities. The traditional tools have the objective to inform and to comply with legal requirements: in other words, they are unidirectional, created by a source and transmitted to a receiver. The modern tools are mainly interactive and iterative, they include a feedback and are implemented and evaluated with quantitative and qualitative methods [1,14]. The quantitative assessment pertains to number of participants in initiatives, acceptance and compliance with interviews and questionnaires, number of media article in press and social media quotes after CISAS press releases, number of contacts and request for information at the website; the qualitative evaluation will be developed by way of interview, focus group, observation and documents providing an account of CISAS project results during its progress [15]. Those efforts provided the significant resources to draft a communication plan for the study, including internal and external communication and a list of evaluation criteria developed with quantitative and qualitative elements; the activities will be also evaluated with specific reports and interviews to participants. Internal communication includes meetings, scientific seminars, and daily activities in remote mode. External communication involves the maintenance of a well-organized website, meetings with authorities, conferences and events, training courses for civil servants, general practitioners and teachers.

### 2.2. Human Biomonitoring (HBM)

Specific attention was devoted to prepare the information needed for the epidemiology component of CISAS project. To implement the HBM and the birth cohort studies human specimens donated by volunteers were requested to detect pollutants (blood, urine, maternal and core blood, placental tissue); they were stored in a biobank or preserved for a period, like all the information collected. Those are peculiar activities in terms of communication, and HBM studies are well-known to cause anxiety in people [16]. It means that all the information material was carefully reviewed, so that all results released observed strict rules and satisfied ethical and legal requirements. The ethical guidelines developed by the International Society for Environmental Epidemiology (ISEE) are very useful in this regard [17]. All study procedures followed the right to know as the basis of self-determination in the use of research results, the distribution of benefits to different groups, the choice to deliver all the results to the research subjects if they want, the identification of tools to reduce exposure, protect volunteers’ health and facilitate the participation in health research and public health decisions. A complete explanation of both project details and research aims was made at enrollment. Moreover, all the subjects were made aware that they may withdraw from participating at any time. In fact, adequate communication of these aspects is useful for the purpose of minimizing difficulties to enrollment and losses during the follow up, in particular aimed at protecting against selection bias [18].

The traditional communication tools are an information sheet, an informed consent to the study including how it will use personal data, a privacy form all-to be signed, and a questionnaire collecting information about lifestyle, diet, health conditions, and potential occupational exposures. This material was tested for comprehension and readability.

The innovative (modern) communication tools included a section of the HBM questionnaire exploring risk perception, access to information on environment and health, and trust in different sources of information [14]. The answers were used to plan the communication of results, to know the media used in the area, to establish positive alliances available among public institutions [16]. The relevant study personnel were either qualified or were given training to select and interview the volunteers and to provide a constant assistance during follow-up in the case of cohort studies.

### 2.3. Public Conferences and Seminars

Public conferences were held to present the project and are linked to project developments and to the involvement of a wider audience and local decision makers to debate specific issues.

Seminars for civil servants and general practitioners were focused on specific issues covered by the CISAS project and open to the wider scientific community. Subjects included marine ecology, biomathematics, environment and health, environmental and epidemiological data in contaminated areas risk communication. The main objective is to provide a general comprehension and more specific insights all along project implementation.

### 2.4. Activities in Schools

The classroom seems like a natural place to teach about the environment and human health [19]. Schoolteachers, being relevant influential actors in the delicate transition phase from school to work, received a specific training activity with the objective of sharing the available scientific knowledge about local environment, and providing instruments for students’ involvement. In recent years, a National Library of Medicine program, aimed to support environmental health education in grades 6–12 in U.S. schools and to engage teachers as collaborators in these activities, has been described. It uses a website as main instrument providing information about environmental health concerns in everyday environments to students in grade 9 and above [19]. CISAS researchers implemented innovative techniques for training courses, to promote active learning by developing practical exercises, building from the experience developed by the Institute of Clinical Physiology of the National Research Council, IFC-CNR, during the coordination of the EU Life Gioconda project. Gioconda, “young citizens count in the decisions on environment and health”, was co-funded by the Directorate General for Environment of the European Commission in 2014–2016 and developed a methodology for peer-to-peer education in environment and health, involving students in local level decision making [20].

(A) Competitions in school. The proposal was to give a prize to classes in primary schools (age 11–13) for photos and/or drawings, and to classes in secondary schools (age 14–19) for short movies that have the theme “environment and health in my city”. The examining commission (5/7 members) used the following reference criteria, which composed an evaluation grid: relevance of competition themes, originality of the subject matter, techniques used (figurative, photography, direction, rhythm, music), effectiveness of communication. A score ranging from 1 to 5, between insufficient and excellent was established; a correction index was used to make comparable ratings of the two types of compositions and of the progressive age of participants. Important international projects support environmental education in schools to inspire future generations to live sustainably [21]. CISAS is using self-produced pictures and movies to improve environmental health literacy of students [22].

(B) A specific training package was dedicated to primary and secondary school teachers, with the aim of enhancing knowledge and awareness about the local reality, the comprehension of environmental pollution phenomena and of impacts on ecosystems and human health. The work developed and the exchanges with teachers was dedicated to understand the transferability of this information to students, finding coincidences with school programs. Several tools were utilized to create an active involvement of students.

(C) A visit on board the Oceanographic Ship L. Sanzo was planned on behalf of limited groups of students to experience water and biota sampling and other environmental investigations. The action aimed at a deep and direct involvement of the students in the CISAS environmental study activities; they will have the opportunity to become familiar with methodological and conceptual approaches used in the different phases of the research.

(D) The creation of a “biodiversity survey form” was proposed to monitor aquatic Phanerogams in the marine environment, to be able to directly observe and recognize some of the typical characteristics of the local environment. The form was prepared in accordance with the Guidelines for the preparation of the Environmental Monitoring Project (PMA) of the works subject to Environmental Impact Assessment (EIA).

(E) A questionnaire on risk perception tested by the Gioconda LIFE project, was proposed to the schools in the CISAS areas, being a component of a methodology to involve young people in discovering their own environmental health issues, while discussing questionnaire results with the CISAS experts [23].

(F) CISAS project finally proposed the creation of a “Didactic Room on environment and health”, having as a reference the best practice of the “Asbestos Room” of the Balbo Institute of Casale Monferrato, an example of a training center for teachers and students, information and involvement for the community, which could be applied to various local environmental and health problems. A set of steps has been defined to explore the availability of school managers to establish a twinning with the Balbo Institute and to implement the project during the coming school year [24].

### 2.5. Media Coverage

The media coverage of CISAS project was organized with the support of the National Research Council press office around each event or in case of specific meetings with authorities. Interaction with the relevant media and personal relationships with more influential people at local level were established.

## 3. Results

### 3.1. Communication Plan

The communication plan was drafted including all the necessary elements for a positive project management and control of the events accomplishment. An information flow, like an excel work plan, was developer to collect the events, to plan the organization of events, the media work and follow up reports, as well as manage a list of indicators to check the improvements and the organization.

### 3.2. Human Biomonitoring (HBM)

The HBM forms, factsheets and questionnaires were used for three HBM studies and the cohort study of newborns implemented in the project areas. The dedicated personnel utilized the tools during enrollment and implementation. A strategy to use and interpret the questionnaires outcomes will permit matching with HBM analyses that are in progress. The global number of results will be around 2500 (1500 adults in the three areas, 1000 mother-baby diads) within the end of year 2020.

### 3.3. Public Conferences and Seminars

After the kick-off meeting in Rome, in October 2016, a conference was held in each location to present the CISAS project. They involved local experts, scholars, researchers and public administrators, to provide information about study design and to initiate a dialogue and exchange of information. From the initial events until May 2019, CISAS organized 7 seminars for a total around 320 experts, 7 public conferences for a total around 950 people, 1 international seminar with around 50 experts; CISAS experts participated to 2 international events in Brussels.

During the seminars, main topics on marine ecology, marine water monitoring, air pollution and human exposure, risk communication, effects of specific pollutants on the human body and their impacts on public health were presented and discussed.

During the public conferences CISAS experts presented the competitions and activities for schools and illustrated the first sets of results produced during the monitoring of sea water, fish and sediments and by an inquiry on food consumption developed with questionnaires and interviews with the managers of large retail chains. The first results were presented in Augusta and later in Crotone, involving experts, civil servants, local authorities and citizen associations. In March 2018 in Augusta, a problem of fish contamination was raised, because the harbor is heavily contaminated by mercury. Fishing is banned, but many people continue to catch and consume local fish that is also sold in the local market. In this case, the news was captured and disseminated by media, raising a strong public attention.

Among the international activities, in November 2018 CISAS organized in Palermo the international open conference “Linking environmental and epidemiological data in contaminated areas” and CISAS experts participated in two international events in Brussels related to the EU Marine Strategy and the integrated assessment of effects of new pollutants.

### 3.4. Activities in Schools

Activity (A) Two competitions for students were realized during the school year 2017–2018 in Milazzo (Valle del Mela area) and Augusta, presented during the launch events of CISAS project: One for photos and/or drawings in primary schools (age 11–13), the second for short movies in secondary schools (age 14–19). The title was: “Environment and health in the Valle del Mela area/Augusta Bay: a complex reality”. In Valle del Mela approximately 200 students, 5 classes of a primary school and 3 classes of a secondary school, entered the competition submitting 12 drawings/photos and 7 videos. In Augusta about 100 students from 4 classes of a primary school and 1 class of a secondary school submitted 4 compositions and one short movie. For each competition, a ranking was made based on the highest score, adding the averages of the scores of each parameter. The commission awarded 3 videos and 3 drawings in Milazzo and 1 video and 3 drawings in Augusta.

The rest of the activities in schools are in progress, starting during the 2019–2020 school-year (the Italian school-year begins in September, end in June of the following year) together with the third competition to be held in Crotone.

Activity (B) The training course is organized in lectures related to: Marine ecology; fate of pollutants in the environment, in food, in the human body; epidemiology, studies about environment and health, statistics and epidemiology, and research on risk perception.

Activity (C) A visit on board of the Oceanographic Ship L. Sanzo is organized on behalf of limited groups of students to experience water and biota sampling and other environmental investigations. A close contact with researchers on board the vessel will allow an exchange of experience and knowledge useful for a more effective understanding of the main objectives of the project.

Activity (D) The “biodiversity survey form” was prepared with the illustration of sampling methodologies, and the final product will be revised and examined with the students during the school year.

Activity (E) The questionnaire will help complete a more detailed picture of risk perception in the areas, both in adults and in children, helpful to understand the exposure to risks, the level of awareness and to better fine-tune the proposals regarding risk control and management measures.

Activity (F) Regarding the “Didactic Room on environment and health” CISAS received a first commitment from a secondary school in Pace del Mela, a Municipality in the Milazzo area, to start building the network and initiating during the coming school year a twining with the “Asbestos Room” in Casale Monferrato [24].

### 3.5. Media Coverage

CISAS is experiencing a fairly good media coverage around the events organized. From 2 to 5 articles have constantly appeared in the local press the day after the conferences and seminars held in project areas, together with around 10 news on web and social media.

## 4. Discussion

The definition of a single model for risk communication to be applied in contaminated sites is not easy, especially if communication is a constitutive element of risk governance [25,26], and complexity, uncertainty, and ambiguity are the characteristics of risk producing potential conflicts [7]. In the three areas (in Sicily and Calabria Regions) where CISAS is in progress, risk governance is still immature, in particular due to the difficult management of highly polluted sites. In such a context, without a coordinated risk governance both the accountability and public debate are difficult to advocate [27].

Project presentations and the first results released demonstrated that the environment and health issue is in the spotlight in the three project locations. Further results, especially regarding HBM activities and the newborn cohort study, will be presented preliminarily to the competent public authorities and the prefect, responsible for public order, and then to the general public during a dedicated conference.

The two competitions in schools not only facilitated a first contact with the local communities, with high visibility on the media and public interest, but they represented an opportunity to discuss the topic of environment and health in the schools. A further competition to be launched in Crotone in the current year will enhance public attention and involve local community.

Public conferences, together with training activities for specific stakeholders will contribute a further enhancement of knowledge, integration of local expertise from different sources, production of recommendations for improving local risk governance. The attention of researchers will then be concentrated to properly present and share the results, showing their significance for implementing protection and prevention actions and for priority settings in remediation activities.

The first results of the environmental monitor presented in Augusta and later in Crotone raised public attention, and consequently the authorities acted establishing more severe controls over the local fishing activities to promote public health. Reading the articles in daily newspapers and social media it is apparent that the close connection and interrelations of environment and health is poorly understood. Accurate and continuous information on the issue will help promote general knowledge and an improvement of habits in the environmental health domain, such as food consumption, exposure to pollutants in water, air and soil and protection of the environment. Producing an accurate information flow is a crucial lesson learnt to be able to prepare the presentation of CISAS final results. Activities to directly involve stakeholders are underway, to follow in particular the phase of results discussion, evaluation and transfer into recommendations for policy implementation at the local level.

A report will summarize CISAS results, it will be distributed to the appropriate institutions and will be available online for the general public. The report will include an executive summary, recommendations for actions and a non-technical summary. It should provide a concise and precise description of the results, their interpretation as well as the experimental conclusions that can be drawn, trying to distinguish between experimental results, to be confirmed, and scientific conclusions connecting environmental pollution, ecosystem changes and human health.

## 5. Conclusions

In this paper the authors presented an overview of the context, the methodology and the activities developed by the CISAS working group dedicated to communication.

The concurrent developments of social media and scientific citizenship contributed changes in legislation and culture, modifying the role of stakeholders in the communication arena. The growth of social media use in fact induced a disintermediation, a never experienced exchange of information among stakeholders and a profound modification in roles and relationships among social actors. Citizens Science encompasses a number of different practices of lay people’s involvement in science, sometimes contributing to formal research, providing new data or developing independent activities; a key feature lays in assuming the responsibility to expand knowledge, to understand scientific language, to enter in the scientific discourse and to be able to use scientific results for decision making [3].

The whole CISAS group of 153 researchers is aware of the crucial importance of communication activities in the framework of a risk governance process, because they call for using the results of the research in direction of evidence-based decisions in the public health protection domain in contaminated sites. Considering the underlying responsibility, the CISAS research group has pursued a specific attention to communication activities involving dedicated personnel and resources, responding to a number of reference principles [14,17]. Respect is the first ethical principle that implies the obligation to produce information comprehensible to everybody, using appropriate language. Transparency and publicity are part of the scientific requirements guaranteeing the reproducibility of research, together with the declaration of conflict of interest. The perspective of research requires impartiality in data management and results production but, operating in the environment and health domain, the choice is to take the part of the public’s health: It means being unbiased (impartial) but not neutral, supporting democracy and recognizing the various responsibilities in the management of risks.

Working to promote citizen science in environment and health research, substantial implications for ethics in health and biomedical science play a central role. Although the research conducted with an observational focus generally raises fewer ethical issues than interventional research, and the CISAS activities presented in this paper are observational, ethical issues related to participatory activities are duly considered, taking into account the rich debate in progress [28].

The support of public participation and mutual listening are natural consequences of the application of the above principles, understood as a promotion of knowledge, competencies and ability to make decisions based on scientific results, regarding the person and the community at large. This implies the involvement of various social sectors and age groups in the different phases of the project; civil servants and public administrators deserve special attention, as well as general practitioners and teachers, as the results of scientific activities should be used in promoting protection and prevention activities. In particular, persuasive results connecting environmental pollution, ecosystem changes and human health will be able to support evidence-based public health interventions in the framework of a transparent risk governance.
